# Fecal DNA methylation markers for detecting stages of colorectal cancer and its precursors: a systematic review

**DOI:** 10.1186/s13148-020-00904-7

**Published:** 2020-08-10

**Authors:** Janhavi R. Raut, Zhong Guan, Petra Schrotz-King, Hermann Brenner

**Affiliations:** 1 Division of Preventive Oncology, German Cancer Research Center (DKFZ) and National Center for Tumor Diseases (NCT), Heidelberg, Germany; 2 Medical Faculty Heidelberg, University of Heidelberg, Heidelberg, Germany; 3 Division of Clinical Epidemiology and Aging Research, German Cancer Research Center (DKFZ), Heidelberg, Germany; 4 German Cancer Consortium (DKTK), German Cancer Research Center (DKFZ), Heidelberg, Germany

**Keywords:** Colorectal cancer, Colorectal adenoma, CRC stage, DNA methylation, Stool, Odds ratio, Risk stratification, Screening biomarker, Stage-specific

## Abstract

**Background:**

DNA methylation biomarkers in stool may have applications in early colorectal cancer (CRC) detection; however, their association with stages of CRC carcinogenesis or their performance in detecting various stages is unclear. We aimed to systematically review the evidence for DNA methylation markers in stool for risk stratification or detection of specific CRC stages, as well as precursors of CRC.

**Methods:**

We conducted a systematic search in line with the Preferred Reporting Items for Systematic Reviews and Meta-Analyses guidelines. We searched PubMed and ISI Web of Knowledge to identify relevant studies published until 14th January 2020. Two reviewers independently extracted data on study population characteristics, candidate genes, methylation measurement methods, odds ratios (ORs), overall and stage-specific sensitivities, specificities, areas under the receiver operating characteristics curve, and *p*-values for statistical significance for OR and for association of methylation levels with stage.

**Results:**

Twenty-seven studies that reported stage-specific associations or performances of fecal DNA methylation markers for detecting colorectal neoplasms were identified. All studies used methylation-specific polymerase chain reaction for assessing methylation levels in the promoter or exon 1 regions of targeted genes. However, most studies were underpowered and limited by their case-control design. Furthermore, the stage-specific associations or sensitivities were validated for two markers (hypermethylation of *GATA4* and *VIM*) only.

**Conclusion:**

Methylation markers in stool may be useful for detection of CRC precursors or CRC staging, but promising candidate markers need to be validated in longitudinal studies on large screening populations, performing epigenome-wide analyses. Identification of stage-specific DNA methylation biomarkers in stool could boost current strategies towards early detection and enable different approaches to precision medicine for CRC.

## Introduction

Worldwide, colorectal cancer (CRC) is the third most common incident cancer and the second leading cause of cancer mortality, accounting for 1.85 million incident cases and ~ 880,000 deaths in 2018 [1]. The disease burden can be decreased with population-based screening, which allows detection of CRC at earlier stages, when chances of cure are substantially higher than at later stages or by detection and removal of precancerous lesions [2–4]. Currently, colonoscopy is the most accurate screening method for early diagnosis of CRC. However, its compliance rate remains very low due to its invasiveness, dietary restriction requirement, and costs [5–7]. While fecal immunochemical test for hemoglobin has been proven to be an effective, currently available non-invasive test to screen patients who are at average risk for the development of CRC, it has limited sensitivity to detect advanced colorectal adenomas (AAs) or stage I CRCs [8, 9]. Thus, effective non-invasive biomarkers that detect early stage CRC and its precursors more reliably are highly desirable.

CRC develops through a multistep process that involves accumulation of both genetic and epigenetic alterations of the cellular genome [10–12]. Among epigenetic modifications, DNA methylation is a common, early, and stable event in tumorigenesis that is easily detectable in small amounts of DNA [13]. Aberrant methylation of an increasing number of genes has been associated with the tumorigenesis of CRC [14–17]. Approval of Cologuard (multi-target stool DNA test that examines *KRAS* mutation, *NDRG4* and *BMP3* methylations, β-actin, plus a hemoglobin immunoassay) [18] and Epi proColon (blood-based test that examines *SEPT9* methylation) [14, 19, 20] by the Food and Drug Administration has further confirmed DNA methylation as an applicable biomarker for CRC screening. Assessing methylation of DNA isolated from stool samples is a biologically rational approach for CRC screening since neoplastic cells are exfoliated into the colonic lumen and are mixed with stool [21, 22]. Several studies have investigated hypermethylation of the cytosine-phosphate-guanine (CpG) islands in gene promoters in stool samples as potential biomarkers for CRC screening [23–28]. In order to facilitate early detection, there is a need to understand the role of aberrant methylation events in each of the stages of colorectal carcinogenesis from non-advanced colorectal adenomas (NAAs) to AAs and then to CRC stages I–IV [29]. However, a comprehensive overview of the associations of these markers with the well-established stages of CRC carcinogenesis or their performance in detecting various stages, specifically early curable colorectal adenomas (Ads), stage I or stage A and stage II or stage B CRC is lacking. The aim of this systematic review is to synthesize results from studies evaluating DNA methylation markers in stool for detecting specific CRC stages, as well as precursors of CRC.

## Materials and methods

We followed the Preferred Reporting Items for Systematic Reviews and Meta-Analyses guidelines [30]; the checklist is shown in Table S1 (see Additional file 1).

### Eligibility criteria

Studies were eligible for inclusion in this systematic review if they met the following inclusion criteria: examining DNA methylation in stool samples from CRC patients at various stages (including at least stage I or II) compared to healthy individuals. Our search was restricted to human research studies in English language. The first step in the selection of eligible studies was based on reading the title and abstract. Articles were excluded if they were (1) not relevant to the topic, (2) not original articles, (3) not based on stool samples, or (4) not assessing methylation markers separately but in combination with genetic markers or immunoassays. Then, the full texts of the remaining articles were read and included when deemed relevant. Finally, studies that did not report stratified results by stage or enough data to calculate them were also excluded.

### Information sources and search strategy

Databases of PubMed and ISI Web of Science were searched for relevant articles until 14th January 2020. Search terms included (colorectal OR colon OR colonic OR rectal OR rectum) AND (cancer OR carcinoma OR adenoma OR neoplasm OR tumor OR malignancy OR serrated OR “sessile serrated”) AND (stool OR fecal OR feces OR faecal OR feacal) AND (“cell-free DNA” OR “cell free DNA” OR “circulating DNA” OR “circulating tumor DNA” OR cfDNA OR cirDNA OR ctDNA OR DNA OR “deoxyribonucleic acid” OR ds-DNA) AND (methylation OR hypermethylation OR hypomethylation) AND (detection OR diagnosis OR screen OR screening OR marker OR biomarker). Additionally, reference lists of relevant studies and reviews were scanned to identify relevant articles. Duplicated hits were removed.

### Data extraction and quality assessment

Two authors (JRR and ZG) independently extracted data from the eligible studies. Extracted variables included first author, publication year, study population (country, numbers of cases and controls, age, and cancer stage distribution), study design, targeted genetic region, DNA methylation assay, and performance in detecting colorectal neoplasms. Data on the following performance-related indicators were extracted: odds ratios (ORs) with 95% confidence intervals (CIs), overall and stage-specific sensitivities, specificity, areas under the receiver operating characteristics curve (AUCs), and *p*-values for statistical significance for OR and for association of methylation with stage. For articles not reporting the measures explicitly, information was extracted from available text and tables to calculate the crude ORs and sensitivities, specificity, and *p*-value for association of methylation level with stage. Discrepancies were discussed and resolved by consensus among the authors.

The quality of included articles was assessed using the QUADAS-2 (Quality Assessment of Diagnostic Accuracy Studies 2) tool [31]. The tool was tailored to the review topic, and the risk of bias and concerns regarding applicability for each study were assessed over four domains: patient selection, index test, reference standard, and flow and timing. The risk of bias and concerns regarding applicability for each study were rated as “High,” “Low,” or “Unclear.” QUADAS-2 assessment was conducted utilizing the Review Manager software, version 5.3 (Copenhagen: The Nordic Cochrane Centre, The Cochrane Collaboration, 2014).

## Results

### Literature search result

**Fig. 1:**
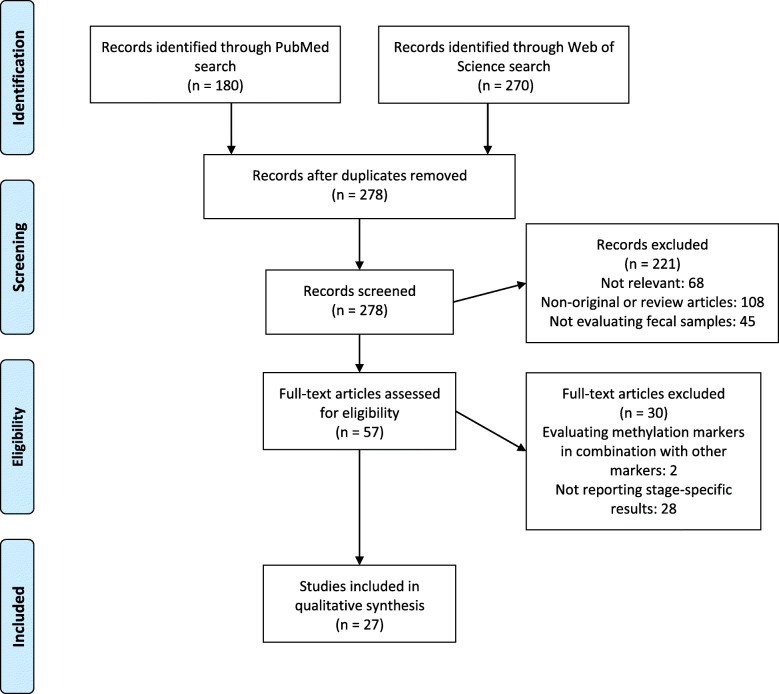
PRISMA flow diagram

The literature search and selection process are shown in Fig. 1. After removing duplicates, 278 articles were identified. On inspection of titles and abstracts, 221 articles were excluded as they were either not relevant or non-original or not evaluating fecal samples. We selected 57 articles for full-text assessment. Of these, two articles were excluded because they evaluated methylation markers in combination with other markers and 28 because they did not report any stage-specific measure of performance or measure of association and did not provide enough data to calculate them. Cross-referencing did not result in identification of any additional studies. Finally, twenty-seven studies met our inclusion criteria and were included in this review. Information on ORs could be extracted or calculated for all studies except one study [32], where it was not possible to calculate the ORs due to lack of information on methylation levels among controls. Overall and stage-specific sensitivities and specificity could be extracted or calculated for all studies. AUCs were reported only in six studies.

### Study characteristics

An overview on the study characteristics is shown in Table S2 (see Additional file 1). The majority of studies were conducted in Asian populations (twenty studies), including thirteen studies from China, five from South Korea, and one each from Japan and Iran. All studies followed a case-control design and collected stool samples from cases at the time of diagnosis or shortly after diagnosis. Only two studies [33, 34] explicitly reported including cases selected in a true screening setting by using samples from patients who underwent colonoscopy for CRC screening. Fifteen studies included Ads, among which six studies included both NAAs and AAs as separate groups, one study included NAAs (but no AAs), and eight studies included AAs (but no NAAs). With regard to CRC stages, six studies investigated all four stages individually, two studies investigated stages I–III individually, one study each investigated stages I–II and stage II–III individually, two studies investigated exclusively stage I, and fifteen studies investigated early (TNM I–II) and late (TNM III–IV) stages. Most of the studies selected controls as participants who were confirmed to have normal findings by colonoscopy, endoscopy, or histology. However, in one study [35] not all controls were verified with colonoscopy. In another study [36], healthy adult volunteers were selected as controls, but it was not reported if they were verified with colonoscopy. Numbers of NAAs ranged from 17 to 41, AAs from 5 to 122, CRCs from 18 to 242, and controls from 16 to 245. Stage-specific numbers of CRC were small in most of the studies. Twelve studies reported the average age and three studies reported the median age. Most of these studies reported a fairly similar age distribution between cases and controls, but a major age difference between cases and controls (52 versus 71 years) was reported in one study [37]. To measure methylation, all studies used methylation-specific polymerase chain reaction (MSP), including nine studies using quantitative methylation-specific real-time polymerase chain reaction (qMSP) and three studies using nested MSP. One study [38] used methylation-specific reverse hybridization assay (MSRH), with MethyLight serving as a reference method. The majority of the studies did not use a validation set to confirm their results. Only four studies [24, 37, 39, 40] performed independent validation of results for some markers to detect CRC, and only two studies [37, 39] validated the stage-specific results in independent populations.

### Overview of fecal DNA methylation markers evaluated for detecting different stages of CRC and its precursors

All studies applied a gene-specific approach and evaluated the methylation status of 25 genes in association with different stages of CRC and its precursors (Table 1). Among these 25 genes, the association of hypermethylation in *COL4A1*, *COL4A2*, *GATA4*, *ITGA4*, *OSMR*, *TLX2*, and *VIM* with CRC risk was further confirmed in independent series of samples from the same studies [24, 37, 39, 40]. Methylation of 12 out of the 25 genes was reported ≥ 2 times and that of the remaining genes was reported only once. Most identified markers were evaluated only individually; eight markers were evaluated only in a panel, and eight markers were evaluated both individually and in a panel. *SFRP2* methylation was assessed most frequently (ten times), followed by *VIM* (five times), and *NDRG4* (four times). The frequency of statistically significant findings for each marker evaluated individually or in a panel ranged from 75 to 100%. All markers were hypermethylated among cases compared to controls. Table S2 (see Additional file 1) presents the targeted genetic region of all markers. Most of the studies assessed methylation levels in the promoter or exon 1 regions of targeted genes.

**Table 1:** Overview of DNA methylation markers in stool evaluated for detection of different stages of colorectal cancer (CRC) and its precursors

Gene	Chromosome	Chen, 2005 [41]	Lenhard, 2005 [23]	Abbaszadegan, 2007 [42]	Itzkowitz, 2008 [39]	Wang, 2008 [43]	Baek, 2009 [44]	Hellebrekers, 2009 [37]	Kim, 2009 [24]	Nagasaka, 2009 [45]	Chang, 2010 [46]	Kalimutho, 2011 [33]	Tang, 2011 [47]	Guo, 2013 [25]	Zhang, 2013 [48]	He, 2014 [26]	Lu, 2014 [49]	Wu, 2014 [36]	Zhang, 2014 [50]	Li, 2015 [27]	Xiao, 2015 [32]	Kriegshäuser, 2017 [38]	Niu, 2017 [51]	Oh, 2017 [35]	Park, 2017 [34]	Yang, 2017 [52]	Han, 2019 [28]	Liu, 2019 [40]	Report frequency	Significant results^a^
*miR-34a*	1																	◊↑											1	1
*ITGA4*	2										□↑																	◊↑	2	2
*TLX2*	2																											∆↑	1	1
*MLH1*	3						□↑																						1	1
*BMP3*	4																								□↑				1	1
*SFRP2*	4					◊↑				◊↑	□↑		◊↑				∆↑		∆↑			◊↑			□↑				10	10
*SNCA*	4																			◊↑						◊↑			2	2
*OSMR*	5								◊↑																				1	1
*TFPI2*	7																								□↑				1	1
*GATA4*	8							◊↑									∆↑												3	3
*SDC2*	8																						◊↑	◊↑			◊↑		3	3
*CDKN2A*	9			◊↑							□↑																		2	2
*MGMT*	10						□↑																						1	1
*VIM*	10	◊↑			◊↑		□↑										∆↑												5	5
*miR-34b/c*	11											◊↑						◊↑											2	2
*WIF-1*	12																		∆↑										2	2
*COL4A1*	13																											◊↑	1	1
*COL4A2*	13																											∆↑	1	1
*ING1*	13															◊↑													1	1
*SPART*	13														◊↑														1	1
*FBN1*	15													◊↑						◊↑									2	2
*NDRG4*	16																∆↑				◊√				□↑				4	3
*HIC1*	17		◊↑																										1	1
*GATA5*	20																∆↑												2	2
*RASSF2*	20									◊↑																			1	1

^a^ Frequency of significant results for association of methylation status of gene with overall CRC risk

◊ Represents markers evaluated only individually

□ Represents markers evaluated only in a panel

∆ Represents markers evaluated both individually and in a panel

↑ Represents hypermethylated marker in CRC cases compared to controls

√ Represents no *p*-value reported or could be calculated

### Overall and stage-wise associations of individual fecal DNA methylation markers with risk of colorectal neoplasms

Twenty-four studies evaluated individual markers, four [24, 37, 39, 40] of which confirmed the findings in independent cohorts (Table 2). The associations of markers with colorectal neoplasms could be evaluated in 23 studies using dichotomized methylation levels quantified by MSP. Only one study [38] used MSRH, with MethyLight serving as a reference method.

**Table 2:** Overall and stage-wise association of fecal DNA methylation markers with risk of colorectal neoplasms

Gene	First author, year, Ref. No.	Country	Study group	N	DNAm assay	OR (95% CI)	*p*-value^a^	CRC stage	N	Stage-specific OR (95% CI)	*p*-value^a^	*p*-value for methylation-stage association^b^
*CDKN2A* (promoter)	Abbaszadegan, 2007 [42]	Iran	CRC Cn	25 20	MSP	11.0 (0.6–212.1)^c^	0.058 0.043^d^	II/B III/C	14 5	4.6 (0.2–120.3)^c^ 57.4 (2.3–1467.3)^c^	0.332 0.001	0.037^e^
*COL4A1* (promoter/exon 1)	Liu, 2019 [40]	China	AA CRC Cn	77 80 83	qMSP	10.3 (4.6–22.9) **57.6 (22.1–150.1)**	< 0.001 **< 0.001**	I/II III/IV	43 37	55.5 (17.7–174.0) 60.2 (17.6–206.1)	< 0.001 < 0.001	1.000
*COL4A2* (promoter/exon 1)	Liu, 2019 [40]	China	AA CRC Cn	77 80 83	qMSP	10.6 (4.3–25.9) **133.9 (43.0–417.2)**	< 0.001 **< 0.001**	I/II III/IV	43 37	105.9 (29.2–383.7) 190.0 (37.5–961.7)	< 0.001 < 0.001	0.815
*FBN1* (promoter)	Guo, 2013 [25]	China	CRC Cn	75 30	MSP	36.0 (7.9–164.7)	< 0.001	I II III IV	12 30 30 3	154.0 (12.7–1875.6) 24.2 (4.8–121.6) 38.5 (7.4–199.9) 28.0 (1.7–458.8)	< 0.001 < 0.001 < 0.001 0.003	0.322
*FBN1* (promoter)	Li, 2015 [27]	China	CRC Cn	89 30	MSP	33.9 (7.5–152.9)^h^	< 0.001	I/A II/B III/C	17 36 36	45.5 (7.4–280.9)^h^ 31.8 (6.4–157.6)^h^ 86.8 (15.6–483.6)^h^	< 0.001 < 0.001 < 0.001	0.237
*GATA4* (promoter)	Hellebrekers, 2009 [37]	Nether-lands	CRC Cn	47 30	qMSP	**14.6 (3.1–68.4)**	**< 0.001**	I/II III/IV	29 17	**13.1 (2.6–65.3)** **20.0 (3.5–112.7)**	**0.000** **< 0.001**	**0.55**^**e**^
*GATA4* (promoter)	Lu, 2014 [49]	China	CRC Cn	56 40	MSP	14.3 (3.1–65)	< 0.001	I/II III/IV	32 24	11.4 (2.3–56.0) 19.0 (3.7–97.1)	0.0006 < 0.001	0.35
*GATA5* (promoter)	Lu, 2014 [49]	China	CRC Cn	56 40	MSP	24.6 (8.3–72.7)	< 0.001	I/II III/IV	32 24	16.8 (5.2–54.2) 51.9 (9.9–273.2)	< 0.001 < 0.001	0.32 0.274^e^
*HIC1* (promoter)	Lenhard, 2005	Germany	AA CRC Cn	13 26 50	MSP	21.8 (2.2–218.0) 35.9 (4.3–301.6)	0.001 < 0.001	I/II III/IV	6 20	98.0 (7.2–1330.0) 16.3 (1.8–151.0)	< 0.001 0.0021	0.138^e^
*ING1* (promoter)	He, 2014 [26]	China	AA CRC Cn	27 61 20	n-MSP	32.3 (3.7–279.3) 53.4 (6.6–432.2)	< 0.001 < 0.001	A/B C/D	33 28	43.7 (5.1–372.8) 69.7 (7.7–631.4)	< 0.001 < 0.001	0.432
*ITGA4* (promoter/exon 1)	Liu, 2019 [40]	China	AA CRC Cn	77 80 83	qMSP	19.0 (5.5–65.4) **125.7 (34.7–456.2)**	< 0.001 **< 0.001**	I/II III/IV	43 37	116.7 (29.2–466.1) 137.8 (32.4–585.4)	< 0.001 < 0.001	0.779
*miR-34a* (promoter)	Wu, 2014 [36]	China	CRC Cn	82 40	MSP	63.0 (13.9–285.7)	< 0.001	I	13	30.4 (5.0–185.5)	< 0.001	0.694
*miR-34b/c* (promoter)	Wu, 2014 [36]	China	CRC Cn	82 40	MSP	1097.2 (59.2–20349.1)^c^	< 0.001	I	13	372.6 (16.7–8322.8)^c^	< 0.001	0.656
*miR-34b/c* (promoter)	Kalimutho, 2011 [33]	Italy	0/ AA CRC Cn	5 23 39	MSP	10.2 (1.4–77.0) 24.5 (6.3–95.9)	0.011 < 0.001	I II III	2 6 3	31.4 (1.3–744.4)^c^ 6.8 (1.1–43.5) 13.6 (1.0–179.0)	0.0032 0.0283 0.0172	0.727^e^
*NDRG4* (promoter)	Xiao , 2015 [32]	China	CRC Cn	84 16	n-MSP	--	--	I/II III/IV	48 36	-- --	-- --	0.209^f^
*NDRG4* (promoter)	Lu, 2014 [49]	China	CRC Cn	56 40	MSP	15.6 (2.0–123.4)	0.001	I/II III/IV	32 24	10.9 (1.3–94.2) 23.4 (2.7–200.9)	0.0098 0.0002	0.200
*OSMR* (promoter)	Kim, 2009 [24]	Belgium	CRC Cn	69 81	qMSP	**11.6 (3.8–35.6)**	**< 0.001**	I II III IV	18 27 18 6	2.4 (0.4–14.3) 24.1 (6.8–84.8) 15.4 (3.9–60.6) 3.9 (0.4–41.2)	0.323 < 0.001 < 0.001 0.236	0.010^e^
*RASSF2: Region 1* (promoter)	Nagasaka, 2009 [45]	Japan	NAA AA Ad CRC Cn	29 27 56 84 113	Hi-SA (n-MSP)	0.5 (0.0–10.7)^c^ 8.3 (1.9–37.5) 3.6 (0.8–15.6) 13.8 (4.0–47.9)	0.678 0.001 0.072 < 0.001	I/II III/IV	40 44	12.2 (3.2–47.2) 15.4 (4.1–57.4)	< 0.001 < 0.001	0.641^f^
*RASSF2: Region 2* (promoter)	Nagasaka, 2009 [45]	Japan	NAA AA Ad CRC Cn	29 27 56 84 113	Hi-SA (n-MSP)	1.3 (0.1–13.1) 2.9 (0.5–18.5) 2.1 (0.4–10.6) 20.4 (6.0–69.7)	0.818 0.234 0.373 < 0.001	I/II III/IV	40 44	10.7 (2.7–41.7) 33.5 (9.2–121.7)	< 0.001 < 0.001	0.015^f^
*SDC2*	Niu, 2017 [51]	China	AA CRC Cn	122 196 179	qMSP	19.4 (9.7–38.5) 59.8 (30.1–118.8)	< 0.001 < 0.001	I/II III/IV	87 109	72.6 (32.0–164.5) 52.0 (24.7–109.6)	< 0.001 < 0.001	0.92 0.373^f^
*SDC2*	Han, 2019 [28]	South Korea	NAA 0/ AA Ad CRC Cn	41 6 47 242 245	LTE-qMSP	3.0 (1.3–6.8) 46.0 (5.2–410.6) 4.3 (2.1–9.1) 83.6 (46.1–151.8)	0.008 < 0.001 < 0.001 < 0.001	I II III IV	55 70 96 21	54.1 (22.9–127.8) 98.2 (38.5–250.7) 79.2 (36.4–172.5) 388.8 (22.8–6619.3)^c^	< 0.001 < 0.001 < 0.001 < 0.001	0.302^e^
*SDC2*	Oh, 2017 [35]	South Korea	NAA CRC Cn	21 50 22	LTE-qMSP	5.0 (0.9–27.7) 90.0 (16.1–503.8)	0.054 < 0.001	I II III IV	12 17 10 11	50.0 (6.1–409.1) 75.0 (9.5–595.1) 90.0 (7.2–1125.5) 188.6 (8.3–4275.8)^c^	< 0.001 < 0.001 < 0.001 < 0.001	0.710^e^
*SFRP2* (promoter)	Kriegshäuser, 2017 [38]	Austria	CRC Cn	2 22	MSRH Methylt	10.0 (2.1–46.6)	0.002	I II	16 18	27.9 (1.1–713.2)^c^ 8.1 (1.7–39.1)	0.01 0.006	0.497^e^
*SFRP2* (promoter)	Zhang, 2014 [50]	China	NAA AA Ad CRC Cn	20 15 35 48 30	MSP	41.5 (2.2–774.6)^c^ 89.2 (4.6–1733.0)^c^ 64.5 (3.7–1137.2)^c^ 78.0 (4.5–1350.0)^c^	0.000 < 0.001 < 0.001 < 0.001	I/A II/B III/C IV/D	7 20 14 7	134.2 (5.6–3192.6)^c^ 61.0 (3.3–1133.8)^c^ 79.8 (4.1–1563.0)^c^ 78.4 (3.5- 1783.6)^c^	< 0.001 < 0.001 < 0.001 < 0.001	0.812^e^
*SFRP2* (promoter)	Lu, 2014 [49]	China	CRC Cn	56 40	MSP	12.0 (3.8–38.3)	< 0.001	I/II III/IV	32 24	9.0 (2.6–31.2) 18.0 (4.7–68.5)	0.0002 < 0.001	0.212
*SFRP2* (promoter)	Tang, 2011 [47]	China	AA CRC Cn	63 169 30	MSP	11.9 (2.6–54.5) 73.6 (16.6–327.5)	0.000 < 0.001	I/II III/IV	99 70	67.5 (14.7–310.8) 84.0 (17.3–409.1)	< 0.001 < 0.001	0.614^f^
*SFRP2: Region 1* (promoter)	Nagasaka, 2009 [45]	Japan	NAA AA Ad CRC Cn	29 27 56 84 113	Hi-SA (n-MSP)	4.4 (1.0–18.6) 11.5 (3.1–41.9) 7.4 (2.3–24.3) 36.3 (12.3–107.8)	0.033 < 0.001 0.000 < 0.001	I/II III/IV	40 44	36.9 (11.4–119.8) 35.9 (11.2–114.7)	< 0.001 < 0.001	0.950^f^
*SFRP2: Region 2* (promoter)	Nagasaka, 2009 [45]	Japan	NAA AA Ad CRC Cn	29 27 56 84 113	Hi-SA (n-MSP)	3.5 (0.9–13.8) 6.2 (1.7–22.1) 4.7 (1.5–14.5) 13.3 (4.9–36.1)	0.066 0.002 0.004 < 0.001	I/II III/IV	40 44	13.0 (4.3–39.0) 13.6 (4.6–40.2)	< 0.001 < 0.001	0.915^f^
*SFRP2* (promoter)	Wang, 2008 [43]	China	AA CRC Cn	34 69 30	qMSP (Methy-Light)	22.6 (4.6–111.2) 93.3 (18.9–460.7)	< 0.001 < 0.001	I/II III/IV	30 39	70.0 (12.5–393.4) 122.5 (20.9–718.2)	< 0.001 < 0.001	0.433 0.488^e^
*SNCA* (promoter)	Yang, 2017 [52]	China	Ad CRC Cn	49 31 64	qMSP	9.2 (3.8–22.6)^g^ 11.3 (3.7–34.8)^g^	< 0.001 < 0.001	I/II III/IV	17 13	14.0 (3.6–55.1) 16.5 (3.3–82.5)	< 0.001 < 0.001	1.000^e^
*SNCA* (promoter)	Li, 2015 [27]	China	CRC Cn	89 30	MSP	138.6 (8.2–2349.8)^c, h^	< 0.001	I/A II/B III/C	17 36 36	107.9 (5.6–2073.1)^c, h^ 239.9 (13.1–4391.9)^c, h^ 119.6 (6.7–2122.1)^c, h^	< 0.001 < 0.001 < 0.001	0.323
*SPART* (promoter)	Zhang, 2013 [48]	China	CRC Cn	96 30	MSP	242.4 (14.2–4142.3)^c^	< 0.001	I	21	117.9 (6.3–2209.1)^c^	< 0.001	0.307
*TLX2* (promoter/exon 1)	Liu, 2019 [40]	China	AA CRC Cn	77 80 83	qMSP	32.0 (9.3–110.2) **210.4 (54.8–807.6)**	< 0.001 **< 0.001**	I/II III/IV	43 37	116.7 (29.2–466.1) 137.8 (32.4–585.4)	< 0.001 < 0.001	0.059
*VIM* (promoter)	Lu, 2014 [49]	China	CRC Cn	56 40	MSP	4.0 (1.4–10.9)	0.006	I/II III/IV	32 24	3.9 (1.3–11.9) 4.1 (1.2–13.3)	0.015 0.018	0.938
*VIM* (promoter)	Itzkowitz, 2008 [39]	USA	CRC Cn	42 241	MSP	**19.6 (8.5–45.2)**	**< 0.001**	I II III IV	11 14 14 3	**46.1 (5.7–369.3)** **27.6 (6.0–128.0)** **8.3 (2.7–26.0)** **31.9 (1.6–629.7)**^**c**^	**< 0.001** **< 0.001** **< 0.001** **0.001**	**0.333**^**e**^
*VIM* (exon 1)	Chen, 2005 [41]	USA	CRC Cn	94 198	MSP	7.5 (4.1–13.9)	< 0.001	I/II III/IV	60 34	6.8 (3.4–13.6) 8.9 (3.9–20.1)	< 0.001 < 0.001	0.533^f^
*WIF-1* (promoter)	Zhang, 2014 [50]	China	NAA AA Ad CRC Cn	20 15 35 48 30	MSP	15.6 (1.7–140.2) 33.1 (3.5–310.3) 24.4 (3.0–199.7) 44.3 (5.6–352.8)	0.003 < 0.001 0.000 < 0.001	I/A II/B III/C IV/D	7 20 14 7	38.7 (3.2–467.8) 67.7 (7.4–617.5) 29.0 (3.1–275.7) 38.7 (3.2–467.8)	0.000 < 0.001 0.000 0.000	0.661^e^

Notes: Stages I/II/III/IV as per Union for International Cancer Control (UICC) classification and stages A/B/C/D as per Dukes classification. Bold fonts represent results from validation set (non-bold fonts represent results without validation)

*Ref.* reference, *No.* number, *DNAm* DNA methylation, *MSP* methylation-specific polymerase chain reaction, *qMSP* quantitative methylation-specific real-time polymerase chain reaction, *n-MSP* nested methylation-specific polymerase chain reaction, *Hi-SA* high-sensitivity assay for bisulfite DNA, *LTE* linear target enrichment, *MSRH* methylation-specific reverse hybridization, *NAA* non-advanced adenoma, *AA* advanced adenoma, *Ad* adenoma, *Cn* control

^a^Statistical significance for OR

^b^Statistical significance for association between methylation level and CRC stage

^c^Calculated using Haldane–Anscombe correction (0.5 added to each cell) [53, 54]

^d^As reported in the article

^e^Calculated using Fisher’s exact test

^f^Calculated using chi-square test

^g^Model adjusted for age and sex

^h^Sum of methylation + stool samples divided by stages is not equal to the total number of methylation + CRC stool samples

Four markers (*SDC2*, *SFRP2*, *SFRP2*: Region 1, *WIF-1*) presented statistically significant associations with NAA, with ORs ranging from 3.0 to 41.5 [28, 45, 50]. Sixteen markers presented statistically significant associations with AA, with ORs ranging from 6.2 to 89.2. For overall adenomas, i.e., a combined group including NAAs and AAs, five markers presented statistically significant associations with ORs ranging from 4.3 to 64.5 [28, 45, 50, 52]. For CRC, 25 markers presented statistically significant positive associations with ORs ranging from 11.0 to 1097.2. In a study by Liu et al. [40], hypermethylation of four candidate genes, namely *COL4A1*, *COL4A2*, *ITGA4*, and *TLX2* was associated with greater susceptibility to CRC in independent cohorts. Furthermore, strong associations of hypermethylation in *GATA4* and *VIM* promoters with CRC risk were found and externally validated in studies by Hellebrekers et al. and Itzkowitz et al. [37, 39], respectively. Only one study [52] reported ORs related to Ads and CRC calculated using multivariable logistic analyses according to methylation levels after adjustment for participants’ age and sex.

When considering analyses by CRC stage, significant associations were estimated for nine, eight, nine, and six hypermethylated markers with stages I (ORs ranging from 27.9 to 372.6), II (ORs ranging from 8.3 to 119.6), III (ORs ranging from 6.8 to 239.9), and IV (ORs ranging from 3.9 to 388.8), respectively. For 11 and 18 hypermethylated markers, significant associations were estimated with early (I/II) stages (ORs ranging from 3.9 to 116.7) and with late (III/IV) stages (ORs ranging from 4.1 to 190.0), respectively. In most of the studies, the methylation-CRC association was stronger when the outcome was restricted to advanced stages than to early stages*.* Only two stage-specific biomarkers (hypermethylation of *GATA4* and *VIM*) were validated in independent samples [37, 39]. Hypermethylation of *GATA4* showed a stronger association with advanced stages than early stages [37]. However, the association of hypermethylation of *VIM* to CRC risk was highest among stage I CRC cases, and no consistent pattern of association according to stage was observed for successive stages [39].

### Overall and stage-wise performance of fecal DNA methylation markers for detection of colorectal neoplasms

An overview of the performance of fecal DNA methylation markers for detection of colorectal neoplasms is shown in Table 3. Sensitivities ranged from 0 to 72%, 7 to 83%, 5 to 76%, and 20 to 94% for identifying NAA, AA, Ad, and CRC, respectively. Specificities ranged from 75 to 100%. AUCs were reported only in six studies and were mostly reported without validation, except one study [40] which reported validated AUCs to discriminate CRC patients from control subjects. Liu et al. [40] reported that methylation levels in the promoter or exon regions of four genes, namely *COL4A1*, *COL4A2*, *ITGA4*, and *TLX2* could differentiate CRC patients from control subjects in independent populations, with AUC values ranging from 0.95 to 0.98. *SDC2* methylation levels evaluated in three studies could discriminate AA patients (44), a combined group including three AA patients along with CRC patients [28] and CRC patients [35, 51] from control subjects, with AUC values of 0.79, 0.90, and 0.92–0.93, respectively. In Yang et al.’s study, *SNCA* methylation levels were reported to discriminate Ad and CRC patients from control subjects, with AUC values of 0.77 and 0.84, respectively [52]. Hellebrekers et al. assessed *GATA4*’s performance in discriminating CRC cases from controls and reported an AUC of 0.81 (sensitivity 71%, specificity 84%) [37]. They further validated this performance in independent samples, resulting in a sensitivity of 51% at a specificity of 93% for identifying CRC cases. The performance of another marker in the promoter region of *VIM* was validated in independent samples, resulting in a sensitivity of 81% at a specificity of 82% for identifying CRC cases [39]. For the remaining markers, validation remains yet to be performed.

**Table 3:** Overall and stage-wise diagnostic performance of individual DNA methylation markers in stool for detection of colorectal neoplasms

Gene	First author, year, Ref. No.	Country	Study group	No.	DNAm assay	Sn (%)	AUC (95%CI), *p*-value	CRC stage	No.	Stage-specific Sn (%)	Specificity (%)
*CDKN2A* (promoter)	Abbaszadegan, 2007 [42]	Iran	CRC Cn	25 20	MSP	20		II/B III/C	14 5	7 60	100
*COL4A1* (promoter/exon 1)	Liu, 2019 [40]	China	AA CRC Cn	77 80 83	qMSP	58 **89**	0.76 (0.69–0.84) **0.97 (0.94–0.99)**	I/II III/IV	43 37	88 89	**88**
*COL4A2* (promoter/exon 1)	Liu, 2019 [40]	China	AA CRC Cn	77 80 83	qMSP	49 **93**	0.78 (0.70–0.85) **0.97 (0.94–0.99)**	I/II III/IV	43 37	91 95	**92**
*FBN1* (promoter)	Guo, 2013 [25]	China	CRC Cn	75 30	MSP	72		I II III IV	12 30 30 3	92 63 73 67	93
*FBN1* (promoter)	Li, 2015 [27]	China	CRC Cn	89 30	MSP	71*		I/A II/B III/C	17 36 36	77* 69* 94*	93
*GATA4* (promoter)	Hellebrekers, 2009 [37]	Netherlands	CRC Cn	28 45	qMSP	71	0.81 (0.70–0.89)	I/II III/IV	18 10	55 100	84
			CRC Cn	47 30	qMSP	**51**		I/II III/IV	29 17	**48** **59**	**93**
*GATA4* (promoter)	Lu, 2014 [49]	China	CRC Cn	56 40	MSP	43		I/II III/IV	32 24	38 50	95
*GATA5* (promoter)	Lu, 2014 [49]	China	CRC Cn	56 40	MSP	84		I/II III/IV	32 24	78 92	83
*HIC1* (promoter)	Lenhard, 2005	Germany	AA CRC Cn	13 26 50	MSP	31 42		I/II III/IV	6 20	67 25	98
*ING1* (promoter)	He, 2014 [26]	China	AA CRC Cn	27 61 20	n-MSP	63 74		A/B C/D	33 28	70 79	95
*ITGA4* (promoter/exon 1)	Liu, 2019 [40]	China	AA CRC Cn	77 80 83	qMSP	42 **83**	0.74 (0.66–0.81) **0.95 (0.92–0.99)**	I/II III/IV	43 37	81 84	**96**
*miR-34a* (promoter)	Wu, 2014 [36]	China	CRC Cn	82 40	MSP	77		I	13	62	95
*miR-34b/c* (promoter)	Wu, 2014 [36]	China	CRC Cn	82 40	MSP	94		I	13	85	100
*miR-34b/c* (promoter)	Kalimutho, 2011 [33]	Italy	0/ AA CRC Cn	5 23 39	MSP	60 78		I II III	2 6 3	100 50 67	87
*NDRG4* (promoter)	Xiao , 2015 [32]	China	CRC Cn	84 16	n-MSP	76		I/II III/IV	48 36	81 69	89
*NDRG4* (promoter)	Lu, 2014 [49]	China	CRC Cn	56 40	MSP	29		I/II III/IV	32 24	22 38	98
*OSMR* (promoter)	Kim, 2009 [24]	Belgium	CRC Cn	69 81	qMSP	**38**		I II III IV	18 27 18 6	11 56 44 17	**95**
*RASSF2: Region 1* (promoter)	Nagasaka, 2009 [45]	Japan	NAA AA Ad CRC Cn	29 27 56 84 113	Hi-SA (n-MSP)	0 19 9 27		I/II III/IV	40 44	25 30	97
*RASSF2: Region 2* (promoter)	Nagasaka, 2009 [45]	Japan	NAA AA Ad CRC Cn	29 27 56 84 113	Hi-SA (n-MSP)	4 7 5 36		I/II III/IV	40 44	23 48	97
*SDC2*	Niu, 2017 [51]	China	AA CRC Cn	122 196 179	qMSP	58 81	0.79 (0.74–0.85) 0.92 (0.89–0.95)	I/II III/IV	87 109	84 79	93
*SDC2*	Han, 2019 [28]	South Korea	NAA 0/ AA Ad CRC Cn	41 6 47 242 245	LTE-qMSP	24 83 32 90	0.90 (0.88–0.93)^#^	I II III IV	55 70 96 21	86 91 90 100	90
*SDC2*	Oh, 2017 [35]	South Korea	NAA CRC Cn	21 50 22	LTE-qMSP	33 90	0.93 (0.85–0.98)	I II III IV	12 17 10 11	83 88 90 100	91
*SFRP2* (promoter)	Kriegshäuser, 2017 [38]	Austria	CRC Cn	18 22	MSRH	61		I II	2 16	100 56	86
*SFRP2* (promoter)	Zhang, 2014 [50]	China	NAA AA Ad CRC Cn	20 15 35 48 30	MSP	55 80 66 56		I/A II/B III/C IV/D	7 20 14 7	71 50 57 57	100
*SFRP2* (promoter)	Lu, 2014 [49]	China	CRC Cn	56 40	MSP	57		I/II III/IV	32 24	50 67	90
*SFRP2* (promoter)	Tang, 2011 [47]	China	AA CRC Cn	63 169 30	MSP	46 84		I/II III/IV	99 70	83 86	93
*SFRP2: Region 1* (promoter)	Nagasaka, 2009 [45]	Japan	NAA AA Ad CRC Cn	29 27 56 84 113	Hi-SA (n-MSP)	14 30 21 57		I/II III/IV	40 44	58 57	97
*SFRP2: Region 2* (promoter)	Nagasaka, 2009 [45]	Japan	NAA AA Ad CRC Cn	29 27 56 84 113	Hi-SA (n-MSP)	14 22 18 38		I/II III/IV	40 44	38 39	96
*SFRP2* (promoter)	Wang, 2008 [43]	China	AA CRC Cn	34 69 30	qMSP (Methy-Light)	62 87		I/II III/IV	30 39	83 90	93
*SNCA* (promoter)	Yang, 2017 [52]	China	NAA AA Ad CRC Cn	36 13 49 31 64	qMSP	72 77 76 84	0.77, < 0.001 0.84, < 0.001	I/II III/IV	17 13	82 85	75
*SNCA* (promoter)	Li, 2015 [27]	China	CRC Cn	89 30	MSP	70*		I/A II/B III/C	17 36 36	65* 81* 67*	100
*SPART* (promoter)	Zhang, 2013 [48]	China	CRC Cn	96 30	MSP	80		I	21	71	100
*TLX2* (promoter/exon 1)	Liu, 2019 [40]	China	AA CRC Cn	77 80 83	qMSP	55 **89**	0.79 (0.71–0.86) **0.96 (0.92–0.99)**	I/II III/IV	43 37	81 97	**96**
*VIM* (promoter)	Lu, 2014 [49]	China	CRC Cn	56 40	MSP	41		I/II III/IV	32 24	41 42	85
*VIM* (promoter)	Itzkowitz, 2008 [39]	USA	CRC Cn	42 241	MSP	**81**		I II III IV	11 14 14 3	**91** **86** **64** **100**	**82**
*VIM* (exon 1)	Chen, 2005 [41]	USA	CRC Cn	94 198	MSP	46		I/II III/IV	60 34	43 50	90
*WIF-1* (promoter)	Zhang, 2014 [50]	China	NAA AA Ad CRC Cn	20 15 35 48 30	MSP	35 53 46 60		I/A II/B III/C IV/D	7 20 14 7	57 70 50 57	97

Notes: Stages I/II/III/IV as per UICC classification and stages A/B/C/D as per Dukes classification. Bold fonts represent results from validation set (non-bold fonts represent results without validation)

*Ref.* reference, *No.* number, *DNAm* DNA methylation, *MSP* methylation-specific polymerase chain reaction, *qMSP* quantitative methylation-specific polymerase chain reaction, *n-MSP* nested methylation-specific polymerase chain reaction, *Hi-SA* high-sensitivity assay for bisulfite DNA, *LTE* linear target enrichment, *MSRH* methylation-specific reverse hybridization, *NAA* non-advanced adenoma, *AA* advanced adenoma, *Ad* adenoma, *Cn* control

*The sum of methylation + stool samples divided by stages is not equal to the total number of methylation + CRC stool samples

^#^For a combined group including three AA (stage 0 CRC) cases along with CRC cases

In stage-specific analyses, numbers of CRC cases in different stages were often small. The efficacy of most of the markers was higher for detecting the late stages compared to the early stages. Stage-specific performances were validated in independent samples for two biomarkers (hypermethylation of *GATA4* and *VIM*) only [37, 39]. Methylated *GATA4* showed a higher sensitivity to detect late stages compared to early stages (59 vs. 48%) at a specificity of 93% [37]. For methylated *VIM*, at a specificity of 82%, reported sensitivity for identifying stage I (91%) was higher than that for identifying stages II (86%) or III (64%). While the highest sensitivity was observed for identifying stage IV CRC cases (100%), the number of cases was very small (three cases) for stage IV [39].

### DNA methylation panels

Combinations of methylation markers as six different panels for detection of colorectal neoplasms were evaluated in six studies, none of which was further validated (Table 4). It was observed that multiple markers combined into a panel showed stronger associations than one marker alone. In Liu et al.’s study [40], when methylation levels of *COL4A2* and *TLX2* were combined, the OR for CRC risk was 422, which was higher than that for methylation levels of *COL4A2* (133.9) and *TLX2* (210), separately. Similarly, in Lu et al.’s study [49], ORs for individual associations of methylated *SFRP2*, *GATA4/5*, *NDRG4*, and *VIM* with CRC ranged from 4.0 to 24.6, but when combined into a panel (defined as presence of at least 1 methylation among the gene promoters), the OR increased to 50.1. In addition, the stage-specific associations were stronger for the panel (with ORs 27.9 and 89.6 for stages I/II and III/IV, respectively) compared to the stage-specific associations of individual markers (ORs ranging from 3.9 to 16.8 and 4.1 to 51.9 for stages I/II and III/IV, respectively). In five out of six studies, stronger associations were observed for advanced stages compared to early stages. However, none of the panels showed a statistically significant difference in methylation levels between stages (*p* > 0.05).

**Table 4:** Overall and stage-wise association of methylation panels in stool with risk of colorectal neoplasms

Gene panel	First author, year, Ref. No.	Country	Study group	No.	DNAm assay	OR (95% CI)	*p*-value^a^	CRC stage	*N*	Stage-specific OR (95% CI)	*p*-value^a^	*p*-value^b^
*MGMT*, *MLH1*, *and VIM* (promoters)	Baek, 2009 [44]	South Korea	NAA AA Ad CRC Cn	30 22 52 60 37	MSP	14.9 (4.4–50.8) 5.3 (1.5–18.8) 9.5 (3.2–28.2) 19.2 (6.3–58.2)	< 0.001 0.01 < 0.001 < 0.001	I/II III/IV	35 25	16.0 (4.9–52.8) 25.6 (6.6–99.7)	< 0.001 < 0.001	0.450
*ITGA4*, *SFRP2*, *and CDKN2A* (promoters)	Chang, 2010 [46]	Korea	NAA AA Ad CRC Cn	17 8 25 30 31	MSP	55.0 (5.9–509.9) 210.0 (11.7–3783.8) 77.1 (8.8–679.2) 70.0 (8.2–594.9)	< 0.001 < 0.001 < 0.001 < 0.001	I/II III/IV	14 16	54.0 (5.6–524.0) 90.0 (9.1–889.9)	< 0.001 < 0.001	0.694^d^
*SFRP2*, *GATA4/5*, *NDRG4*, *and VIM* (promoters)	Lu, 2014 [49]	China	CRC Cn	56 40	MSP	50.1 (10.6–237.1)	< 0.001	I/II III/IV	32 24	27.9 (5.8–134.2) 89.6 (5.1–1583.0)^c^	< 0.001 < 0.001	0.501^d^
*SFRP2 and WIF-1* (promoters)	Zhang, 2014 [50]	China	NAA AA Ad CRC Cn	20 15 35 48 30	MSP	35.4 (4.0–313.4) 116.0 (10.9–1229.9) 55.6 (6.7–459.5) 125.7 (15.1–1048.2)	< 0.001 < 0.001 < 0.001 < 0.001	I/A II/B III/C IV/D	7 20 14 7	174.0 (9.5–3187.4) 116.0 (11.9–1128.2) 72.5 (7.2–727.6) 295.0 (10.9–7994.2)^c^	< 0.001 < 0.001 < 0.001 < 0.001	0.605^d^
*SFRP2*, *TFPI2*, *NDRG4*, *and BMP3* (promoters)	Park, 2017 [34]	South Korea	AA CRC Cn	36 35 40	MSP	3.2 (1.2–8.3) 20.2 (4.3–95.7)	0.017 < 0.001	I/II III/IV	17 18	9.2 (1.9–45.5) 45.0 (2.5–798.2)^c^	0.003 0.000	0.229^d^
*COL4A2 and TLX2* (promoter/exon 1)	Liu, 2019 [40]	China	AA CRC Cn	77 80 83	qMSP	43.8 (10.0–190.9) 422.4 (85.0–2098.2)	< 0.001 < 0.001	I/II III/IV	43 37	249.8 (48.1–1296.4) 1458.0 (128.1–16,600.2)	< 0.001 < 0.001	0.168

Note: Stages I/II/III/IV as per UICC classification and stages A/B/C/D as per Dukes classification

*Ref.* reference, *No.* number, *DNAm* DNA methylation, *MSP* methylation-specific polymerase chain reaction, *qMSP* quantitative methylation-specific real-time polymerase chain reaction, *n-MSP* nested methylation-specific polymerase chain reaction, *Hi-SA* high-sensitivity assay for bisulfite DNA, *LTE* linear target enrichment, *MSRH* methylation-specific reverse hybridization, *NAA* non-advanced adenoma, *AA* advanced adenoma, *Ad* adenoma, *Cn* control

^a^Statistical significance for OR

^b^Statistical significance for association between methylation level and CRC stage

^c^Calculated using Haldane–Anscombe correction (0.5 added to each cell) [53, 54]

^d^Calculated using Fisher’s exact test

Regarding the screening performances, sensitivities ranged from 55 to 70%, 46 to 88%, 60 to 72%, and 70 to 96% for NAA, AA, Ad, and CRC, respectively. Stage-specific sensitivities ranged from 64 to 94% and 75 to 100% for stages I/II and III/IV, respectively (Table 5). Specificities ranged from 55 to 98%. In stage-specific analyses, most of the studies showed that efficacy in detecting advanced stages of CRC was better than in early stage of CRC.

**Table 5:** Overall and stage-wise performance of methylation panels in stool for detection of colorectal neoplasms

Gene panel	First author, year, Ref. No.	Country	Study group	No.	DNA methylation assay	Sn (%)	CRC stage	No.	Stage-specific Sn (%)	Sp (%)
*MGMT*, *MLH1*, *and VIM* (promoters)	Baek, 2009 [44]	South Korea	NAA AA Ad CRC Cn	30 22 52 60 37	MSP	70 46 60 75	I/II III/IV	35 25	71 80	87
*ITGA4*, *SFRP2*, *and CDKN2A* (promoters)	Chang, 2010 [46]	Korea	NAA AA Ad CRC Cn	17 8 25 30 31	MSP	65 88 72 70	I/II III/IV	14 16	64 75	97
*SFRP2*, *GATA4/5*, *NDRG4 and VIM* (promoters)	Lu, 2014 [49]	China	CRC Cn	56 40	MSP	96	I/II III/IV	32 24	94 100	65
*SFRP2 and WIF-1* (promoters)	Zhang, 2014 [50]	China	NAA AA Ad CRC Cn	20 15 35 48 30	MSP	55 80 66 81	I/A II/B III/C IV/D	7 20 14 7	86 80 71 100	97
*SFRP2*, *TFPI2*, *NDRG4*, *and BMP3* (promoters)	Park, 2017 [34]	South Korea	AA CRC Cn	36 35 40	MSP	72 94	I/II III/IV	17 18	88 100	55
*COL4A2 and TLX2* (promoter/exon 1)	Liu, 2019 [40]	China	AA CRC Cn	77 80 83	qMSP	52 91	I/II III/IV	43 37	86 97	98

Note: Stages I/II/III/IV as per UICC classification and stages A/B/C/D as per Dukes classification

*Ref*. reference, *No*. number, *Sn* sensitivity, *Sp* specificity, *MSP* methylation-specific polymerase chain reaction, *qMSP* quantitative methylation-specific real-time polymerase chain reaction, *NAA* non-advanced adenoma, *AA* advanced adenoma, *Ad* adenoma, *Cn* Control

### Methylation levels of individual markers or panels by CRC stage

Nine studies reported on the association between methylation levels of individual markers or panels and CRC stage (Tables 2 and 4). For the remaining studies, we evaluated the association between methylation levels of individual markers or panels and CRC stage using chi-square or Fisher’s exact test. A significant stepwise increase in methylation levels of *CDKN2A* (*p* = 0.04) [42], *OSMR* (*p* = 0.01) [24], and *RASSF2*: Region 2 (*p* = 0.02) [45] promoters with stage was observed, with higher levels in advanced stage patients compared with early stage patients. For the remaining individual markers or panels, the difference in methylation levels between stages was not statistically significant (*p* > 0.05).

### Quality assessment of studies

The results for the quality assessment of studies using the QUADAS tool are presented in Figures S1 and S2 (see Additional file 1). The greatest potential risk of bias came from patient selection as all but two studies selected participants in clinical settings rather than screening settings. Most studies (23/27) were rated as having unclear risk of bias for the index test due to lack of information on whether a pre-specified threshold was used and interpretation of results without conducting validation studies. For most of the studies, there was no concern of bias for the reference standard. The risk of bias for flow and timing was low for 20 studies and unclear for 7 studies. Applicability concerns were high for patient selection as most of the studies collected blood samples from symptomatic cases at the time of diagnosis rather than evaluating samples of participants recruited in true screening settings.

## Discussion

Identification of stage-specific DNA methylation biomarkers in stool could boost current screening strategies towards early-stage detection and enable different approaches to precision medicine for CRC. In this systematic literature review, we identified twenty-seven studies evaluating twenty-five fecal methylation markers for detection of different stages of CRC and its precursors. The most frequently used platform for assessing methylation was MSP with various modifications. Multiple methylation markers showed significant associations with NAA, AA, Ad, TNM stages I–IV, and overall CRC in either univariate or multivariate regression analysis. A majority of the studies showed that the efficacy of either single methylation biomarkers or biomarker panels was higher for detecting advanced CRC stages compared to early CRC stages. However, most of the studies had relatively small sample sizes which might have limited the assessment of efficacy to detect colorectal neoplasms at different stages. Furthermore, most of the findings according to cancer stage lacked any form of validation.

We identified twenty-four studies reporting stage-specific results for individual markers. In a study by Guo et al. [25], a promising marker for stage I CRC detection was identified in the promoter region of *FBN1*. The marker showed a 92% sensitivity at 93% specificity (OR = 154.0, 95% CI = 12.7–1875.6, *p* < 0.001) for detecting stage I CRC. Another marker in the gene *SDC2* reported by Han et al. [28] showed a promising performance for stage II CRC detection with 91% sensitivity at 90% specificity (OR = 98.2, 95% CI = 38.5–250.7, *p* < 0.001). However, these findings should be interpreted with caution, considering that they were from studies with relatively small number of stage-specific cases and are yet to be validated in larger independent samples. Validation in an independent cohort was performed in a study by Itzkowitz et al. [39], resulting in the identification of a promising marker in the promoter region of *VIM*. While the stage-specific numbers of cases were small (3-14), the marker showed high sensitivities of 91%, 86%, 64%, and 100% for stages I–IV respectively at 82% specificity in the validation set.

The majority of findings in this review came from case control studies in different populations that varied in quality. A key feature of diagnostic biomarker identification studies is that the participants should reflect the screening population and the recruitment conditions for cases, and controls should be fully comparable. However, the studies mostly recruited participants in clinical settings rather than screening settings, introducing the potential of selection bias that might have influenced the results. Of the twenty-seven reviewed studies, twenty studies were conducted in Asian populations, limiting the overall generalizability of the results. Furthermore, the relatively small size of most of these studies resulted in inadequate power to estimate stage-specific results in stratified analyses. When selecting cases, of the twenty-seven reviewed studies, only fourteen studies included AAs which have a high risk of transformation to cancer and would be most relevant to stratify risk for developing CRC. In order to identify promising stage-specific biomarkers for CRC screening, there is a need for large-scale prospective screening cohorts from populations that are diverse with respect to geography, age, and sex.

Regarding sample collection, storage, and pre-treatment steps for methylation assays, we found that a substantial heterogeneity existed among the included studies. This may have contributed, in part, to inconsistent DNA methylation measurements. Although the advantages of adding stabilization buffer to stool samples during collection (to guarantee the most consistent yield in human DNA) have been effectively demonstrated [55], not all studies reported using a preservative buffer while sample collection. There was great variation (30 min–12 h after defecation) in the time intervals between completed collection and placement of the samples in the laboratory freezer, where they were frozen at varying temperatures (− 80 to 4 °C) until DNA extraction. Only some studies reported centrifuging the samples before freezing. The studies also used different amounts of starting material (180–300 mg) and kits for extracting DNA from samples including QIAamp DNA Stool Mini Kit, TIANamp Genomic DNA kit, Stool DNA Extraction kit (Bioneer Corporation), and precipitation DNA pellet by centrifugation. DNA concentration was measured in some studies using Qubit dsDNA BR assay kit or ultraviolet spectrophotography. Few studies verified the quality of DNA by agarose gel electrophoresis and polymerase chain reaction (PCR) amplification of the human β-actin. Furthermore, the studies used varying amounts (500 ng–2 μg) of genomic DNA for bisulfite conversion using different kits including EZ DNA Methylation-Gold kit (ZYMO Research, USA) and EpiTect Bisulfite Kit (Qiagen). Bisulfite-converted DNA was either used immediately for methylation analysis or stored at − 20 °C until further use. While template amount of DNA in the PCR is the largest contributor of technical variability in bisulfite PCR-based DNA methylation analyses, storage of bisulfite converted DNA, DNA input in the bisulfite conversion reaction, and type of bisulfite kit also contribute to various degrees of variability [56]. Thus, standardization of these methodological approaches is critical to improve the reliability of findings and replication across studies.

The selection of the genomic regions to be studied is one of the critical challenges to establishing DNA methylation biomarkers that are clinically useful. An unbiased genome-wide screening approach would help discover novel sites and genes of interest. So far, all studies have been based on candidate gene approaches and very few studies investigated DNA methylation in the same gene. Assessment of DNA methylation in all studies was using MSP, which interrogates only a few CpGs (in the gene promoters in most of the studies), serving as a surrogate for the methylation status of the whole region. As methylation patterns often vary largely across genomic regions and are poorly defined [57, 58], analysis of sequence stretches with multiple CpGs (small groups of CpGs (CpG units), regional methylation changes, or site clusters) is less informative compared to analysis at single nucleotide resolution (quantification of methylation for individual CpGs). Furthermore, to make it easier to transfer technologies to different labs and to create clinical standards, the use of methods that deliver quantitative methylation data is desirable. Among the reviewed studies, fifteen studies used conventional MSP, three studies used nested MSP, and nine studies used qMSP. Although MSP is a highly sensitive method, especially when nested PCR approaches are used, it is not quantitative and bears a significant risk of false-positive results and variability of results due to assay conditions (e.g., primer design, annealing temperature, cycle number) [59–61]. While a few studies used qMSP which is highly specific and more sensitive than conventional PCR, the method still provides a low coverage of CpGs [61, 62]. Given the availability of new high-throughput technologies that are able to investigate DNA methylation in a genome-wide manner, future studies should make use of these to allow for unbiased methylation analysis of a large number of CpGs.

A critical factor affecting the clinical utility of a molecular biomarker is specificity. Low specificity results in high numbers of false-positive results, exposing the patient to unnecessary invasive evaluation, with adverse consequences and increased cost of care. It is well recognized that methylation biomarkers, particularly those identified using non-quantitative MSP-based methodologies and poorly controlled enzyme-based approaches are prone to generating false-positive results [59–61]. The most well-studied blood-based screening marker SEPT9 methylation has also been associated with false-positive results [63, 64]. Since most of the reviewed studies used conventional MSP, these results must be very carefully interpreted. Replication of the significant findings in future studies is of paramount importance in order to limit the number of false-positives. Specificity of methylation markers could be improved by studying the biological role of a biomarker and considering tissue-specific methylation patterns in the gene of interest to distinguish the truly cancer-related aberrant methylation event from baseline methylation.

Compared to molecular markers such as methylation of *SEPT9* [19, 65, 66] in tumor-derived cell-free DNA, microRNA signatures in various biofluids (plasma, serum, or stool) [67–69], genetic [70, 71], or proteomic markers [72–74] performance of methylation markers in stool DNA for detecting various stages of CRC seems poorer. In the reviewed studies, methylation of several genes was associated with increased risk of NAA/AA/Ad, early and late stages of CRC, supporting a role of DNA methylation at all stages of CRC, and suggesting potential use of these biomarkers for risk stratification in CRC screening. Nevertheless, evidence is lacking on whether the methylation-CRC association could vary by cancer stage. Stronger methylation-CRC association in the few cases of advanced stages than early stages should be interpreted with caution considering that they are from small-scale studies with cross-sectional data. Large-scale prospective studies conducted in true screening settings are needed before we can draw an inference that, for example, these markers are measurable in early and late stages of colorectal carcinogenesis. Furthermore, more studies should provide stage-specific analyses to explore the relationship of gene-specific methylation with various stages of CRC.

This review offers a comprehensive overview of all diagnostic stool DNA methylation biomarkers investigated in separate stages of colorectal carcinogenesis from NAA to CRC stages I–IV. We comprehensively extracted all relevant information from twenty-seven identified studies and completed a quality assessment using a recognized evaluation tool [31]. Our review has several limitations. Despite comprehensive search in two well-established databases and cross-referencing, it is possible that some relevant studies were missed, especially those published in languages other than English. Several studies had to be excluded in full text review because they did not report any stage-specific measure of diagnostic performance or measure of association and did not provide enough data to calculate them. Such selective reporting could have introduced an outcome reporting bias [75]. We could not combine the results of independent studies as a meta-analysis due to a substantial heterogeneity existing across the reviewed studies. Finally, associations between methylation markers in stool and stage of colorectal carcinogenesis cannot be established from the reviewed studies.

## Conclusion

Our review suggests the possibility of using stool-based methylation markers for risk stratification or stage-specific detection of CRC and its precursors, as a number of studies support an association between methylation changes in stool and different stages of CRC. A number of promising methylation markers have been reported, but optimized stage-specific markers are yet to be developed and promising candidate markers need to be validated in prospective study cohorts and tested in large screening populations by well-designed studies. While the investigation of methylation changes in stool DNA holds great promises, future studies should apply more standardized methods and use unbiased genome-wide methylation analyses to define a consistent panel of stool DNA methylation biomarkers for CRC early detection and staging. This work will further the research into clinically useful biomarkers and could potentially lead not only to concise and timely diagnosis of CRC, but possibly to the discovery of predictive markers associated with CRC stages.

## Supplementary information

**Additional file 1: Table S1**. PRISMA 2009 Checklist. **Table S2.** Study characteristics of reviewed studies. **Figure S1.** Risk of bias and applicability concerns graph: review authors’ judgements about each domain presented as percentages across included studies. **Figure S2.** Risk of bias and applicability concerns summary: review authors' judgements about each domain for each included study.

## Data Availability

All data collected, generated, or analyzed during this study are included in this published article, or available as its additional file.

## Abbreviations

AA: Advanced colorectal adenoma
Ad: Colorectal adenoma
AUC: Area under the receiver operating characteristics curve
CI: Confidence intervals
CpG: Cytosine-phosphate-guanine
CRC: Colorectal cancer
MSP: Methylation-specific polymerase chain reaction
MSRH: Methylation-specific reverse hybridization assay
NAA: Non-advanced colorectal adenoma
OR: Odds ratio
qMSP: Quantitative methylation-specific real-time polymerase chain reaction
QUADAS: Quality Assessment of Diagnostic Accuracy Studies
