# Supplementary material for: Fecal DNA methylation markers for detecting stages of colorectal cancer and its precursors: a systematic review
Source: Clin Epigenetics. 2020 Aug 10;12:122. doi: 10.1186/s13148-020-00904-7 (PMC7418412; doi:10.1186/s13148-020-00904-7)
Supplement: Supplementary file 1 — Additional file 1: Table S1. PRISMA 2009 Checklist. Table S2. Study characteristics of reviewed studies. Figure S1. Risk of bias and applicability concerns graph: review authors’ judgements about each domain presented as percentages across included studies. Figure S2. Risk of bias and applicability concerns summary: review authors' judgements about each domain for each included study. [file 13148_2020_904_MOESM1_ESM.docx]

Table S1. PRISMA 2009 Checklist

| **Section/topic** | | **#** | | **Checklist item** | | **Reported on page #** | |
| --- | --- | --- | --- | --- | --- | --- | --- |
| **TITLE** | | | | | |  | |
| Title | | 1 | | Identify the report as a systematic review, meta-analysis, or both. | | 1 | |
| **ABSTRACT** | | | | | |  | |
| Structured summary | | 2 | | Provide a structured summary including, as applicable: background; objectives; data sources; study eligibility criteria, participants, and interventions; study appraisal and synthesis methods; results; limitations; conclusions and implications of key findings; systematic review registration number. | | 2 | |
| **INTRODUCTION** | | | | | |  | |
| Rationale | | 3 | | Describe the rationale for the review in the context of what is already known. | | 3, 4 | |
| Objectives | | 4 | | Provide an explicit statement of questions being addressed with reference to participants, interventions, comparisons, outcomes, and study design (PICOS). | | 4 | |
| **METHODS** | | | | | |  | |
| Protocol and registration | | 5 | | Indicate if a review protocol exists, if and where it can be accessed (e.g., Web address), and, if available, provide registration information including registration number. | | 4 | |
| Eligibility criteria | | 6 | | Specify study characteristics (e.g., PICOS, length of follow-up) and report characteristics (e.g., years considered, language, publication status) used as criteria for eligibility, giving rationale. | | 4, Table S1 | |
| Information sources | | 7 | | Describe all information sources (e.g., databases with dates of coverage, contact with study authors to identify additional studies) in the search and date last searched. | | 5 | |
| Search | | 8 | | Present full electronic search strategy for at least one database, including any limits used, such that it could be repeated. | | 5 | |
| Study selection | | 9 | | State the process for selecting studies (i.e., screening, eligibility, included in systematic review, and, if applicable, included in the meta-analysis). | | 4, 5 | |
| Data collection process | | 10 | | Describe method of data extraction from reports (e.g., piloted forms, independently, in duplicate) and any processes for obtaining and confirming data from investigators. | | 5 | |
| Data items | | 11 | | List and define all variables for which data were sought (e.g., PICOS, funding sources) and any assumptions and simplifications made. | | 5, 6 | |
| Risk of bias in individual studies | | 12 | | Describe methods used for assessing risk of bias of individual studies (including specification of whether this was done at the study or outcome level), and how this information is to be used in any data synthesis. | | 6 | |
| Summary measures | | 13 | | State the principal summary measures (e.g., risk ratio, difference in means). | | 5, 6 | |
| Synthesis of results | | 14 | | Describe the methods of handling data and combining results of studies, if done, including measures of consistency (e.g., I^2^) for each meta-analysis. | | -- | |
| **Section/topic** | | **#** | | **Checklist item** | | **Reported on page #** | |
| Risk of bias across studies | | 15 | | Specify any assessment of risk of bias that may affect the cumulative evidence (e.g., publication bias, selective reporting within studies). | | -- | |
| Additional analyses | | 16 | | Describe methods of additional analyses (e.g., sensitivity or subgroup analyses, meta-regression), if done, indicating which were pre-specified. | | -- | |
| **RESULTS** | | | | | |  | |
| Study selection | | 17 | | Give numbers of studies screened, assessed for eligibility, and included in the review, with reasons for exclusions at each stage, ideally with a flow diagram. | | 6, Figure 1 | |
| Study characteristics | | 18 | | For each study, present characteristics for which data were extracted (e.g., study size, PICOS, follow-up period) and provide the citations. | | 7, 8,  Table S1 | |
| Risk of bias within studies | | 19 | | Present data on risk of bias of each study and, if available, any outcome level assessment (see item 12). | | 16, Figures S1, S2 | |
| Results of individual studies | | 20 | | For all outcomes considered (benefits or harms), present, for each study: (a) simple summary data for each intervention group (b) effect estimates and confidence intervals, ideally with a forest plot. | | 8- 16 | |
| Synthesis of results | | 21 | | Present results of each meta-analysis done, including confidence intervals and measures of consistency. | | -- | |
| Risk of bias across studies | | 22 | | Present results of any assessment of risk of bias across studies (see Item 15). | | -- | |
| Additional analysis | | 23 | | Give results of additional analyses, if done (e.g., sensitivity or subgroup analyses, meta-regression [see Item 16]). | | -- | |
| **DISCUSSION** | | | | | |  | |
| Summary of evidence | | 24 | | Summarize the main findings including the strength of evidence for each main outcome; consider their relevance to key groups (e.g., healthcare providers, users, and policy makers). | | 17- 21 | |
| Limitations | | 25 | | Discuss limitations at study and outcome level (e.g., risk of bias), and at review-level (e.g., incomplete retrieval of identified research, reporting bias). | | 21, 22 | |
| Conclusions | | 26 | | Provide a general interpretation of the results in the context of other evidence, and implications for future research. | | 22 | |
| **FUNDING** | | | | | |  | |
| Funding | | 27 | | Describe sources of funding for the systematic review and other support (e.g., supply of data); role of funders for the systematic review. | | 34 | |

Table S2. Study characteristics of reviewed studies

| First author,  year,  Ref. No. | Country | Study  Design | Characteristics of Cn | Study  Group | No. | Age (year) | CRC stage | N | DNAm  assay | Gene (region) |
| --- | --- | --- | --- | --- | --- | --- | --- | --- | --- | --- |
| Chen, 2005 (1) | USA | Case-control | Negative for colon cancer on colonoscopic exam | CRC  Cn | 94  198 | __ | I/ II  III/ IV | 60  34 | MSP | *VIM (exon 1)* |
| Lenhard, 2005 (2) | Germany | Case-control | Endoscopically normal | AA  CRC  Cn | 13  26  50 | 68.6  65.8  57.7 | I/ II  III/ IV | 6  20 | MSP | *HIC1 (promoter)* |
| Abbaszadegan, 2007 (3) | Iran | Case-control | Normal colonoscopy | CRC  Cn | 25  20 | __ | II/B  III/C | 14  5 | MSP | *CDKN2A (promoter)* |
| Itzkowitz, 2008 (4) | USA | Case-control | Normal colonoscopy | CRC  Cn | 42  241 | 67.4  56.9 | I  II  III  IV | 11  14  14  3 | MSP | *VIM (promoter)* |
| Wang, 2008 (5) | China | Case-control | Endoscopically normal | AA  CRC  Cn | 34  69  30 | __ | I/ II  III/ IV | 30  39 | qMSP  (Methy-Light) | *SFRP2 (promoter)* |
| Baek, 2009 (6) | South  Korea | Case-control | Endoscopically normal | NAA  AA  Ad  CRC  Cn | 30  22  52  60  37 | 58.5  61.4  58.8 | I/ II  III/ IV | 35  25 | MSP | *MGMT (promoter)*  *MLH1 (promoter)*  *VIM (promoter)* |
| Hellebrekers, 2009 (7) | Nether-lands | Case-control | Normal colonoscopy | CRC  Cn | 47  30 | 71  52 | I/ II  III/ IV | 29  17 | qMSP | *GATA4 (promoter)* |
| Kim, 2009 (8) | Belgium | Case-control | Normal colonoscopy | CRC  Cn | 69  81 | __ | I  II  III  IV | 18  27  18  6 | qMSP  (TaqMan) | *OSMR (promoter)* |
|  |  |  |  |  |  |  |  |  |  |  |
| Nagasaka, 2009 (9) | Japan | Case-control | Subjects without neoplastic or active diseases who underwent colonoscopy and gastroduodenal endoscopy | NAA  AA  Ad  CRC  Cn | 29  27  56  84  113 | 62.7  66.7  64.6  65.2  66.1 | I/ II  III/ IV | 40  44 | High-sensitivity assay for bisulfite DNA  (n-MSP) | *RASSF2 Region 1 and 2 (promoter)*  *SFRP2 Region 1 and 2 (promoter)* |
| Chang, 2010 (10) | South  Korea | Case-control | Endoscopically normal | NAA  AA  Ad  CRC  Cn | 17  8  25  30  31 | 60.1  61.7  58.8 | I/ II  III/ IV | 14  16 | MSP | *ITGA4 (promoter)*  *SFRP2 (promoter)*  *CDKN2A (promoter)* |
| Kalimutho, 2011 (11) | Italy | Case-control | Normal colonoscopy | 0/ AA  CRC  Cn | 5  23  39 | 66^#,^ *  58^#^ | I  II  III | 2  6  3 | MSP | *miR-34b/c (promoter)* |
| Tang, 2011 (12) | China | Case-control | Macroscopically normal colorectal mucosa | AA  CRC  Cn | 63  169  30 | __ | I/ II  III/ IV | 99  70 | MSP | *SFRP2*  *(promoter: -219 to -81 relative to the transcription start site)* |
| Guo, 2013 (13) | China | Case-control | Endoscopically/ histologically normal | CRC  Cn | 75  30 | 58.5  58.4 | I  II  III  IV | 12  30  30  3 | MSP | *FBN1 (promoter)* |
| Zhang, 2013 (14) | China | Case-control | Endoscopically normal | CRC  Cn | 96  30 | 62.2  62.9 | I | 21 | MSP | *SPART (promoter)* |
| He, 2014 (15) | China | Case-control | Endoscopically normal | AA  CRC  Cn | 27  61  20 | __ | A/ B  C/ D | 33  28 | n-MSP | *ING1 (promoter)* |
|  |  |  |  |  |  |  |  |  |  |  |
| Lu, 2014 (16) | China | Case-control | Endoscopically normal | CRC  Cn | 56  40 | 60.6  59.8 | I/ II  III/ IV | 32  24 | MSP | *GATA4 (promoter)*  *GATA5 (promoter)*  *NDRG4 (promoter)*  *SFRP2 (promoter)*  *VIM (promoter)* |
| Wu, 2014 (17) | China | Case-control | Healthy adult volunteers | CRC  Cn | 82  40 | __ | I | 13 | MSP | *miR-34a (promoter)*  *miR-34b/c (promoter)* |
| Zhang, 2014 (18) | China | Case-control | Endoscopically normal | NAA  AA  Ad  CRC  Cn | 20  15  35  48  30 | __ | I/ A  II/ B  III/ C  IV/ D | 7  20  14  7 | MSP | *SFRP2 (promoter)*  *WIF-1 (promoter)* |
| Li, 2015 (19) | China | Case-control | Normal colonoscopy | CRC  Cn | 89  30 | __ | I/A  II/B  III/C | 17  36  36 | MSP | *FBN1 (promoter)*  *SNCA (promoter)* |
| Xiao, 2015 (20) | China | Case-control | Normal pathology | CRC  Cn | 84  16 | 55.6 | I/ II  III/ IV | 48  36 | n-MSP | *NDRG4 (promoter)* |
| Kriegshäuser, 2017 (21) | Austria | Case-control | Endoscopically normal | CRC  Cn | 18  22 | __ | I  II | 16  18 | qMSP & Methylation specific reverse hybridization | *SFRP2 (promoter)* |
| Niu, 2017 (22) | China | Case-control | Normal colonoscopy | AA  CRC  Cn | 122  196  179 | 61^#^  61^#^  56^#^ | I/ II  III/ IV | 87  109 | qMSP | *SDC2* |
| Oh, 2017 (23) | South Korea | Case-control | Healthy subjects | NAA  CRC  Cn | 21  50  22 | 63.4  61.9  58.8 | I  II  III  IV | 12  17  10  11 | meSDC2 LTE-qMSP | *SDC2* |
| Park,  2017 (24) | South  Korea | Case-control | Endoscopically normal | AA  CRC  Cn | 36  35  40 | 63.2  60.6  55.7 | I/ II  III/ IV | 17  18 | MSP | *SFRP2 (promoter)*  *TFPI2 (promoter)*  *NDRG4 (promoter)*  *BMP3 (promoter)* |
| Yang, 2017 (25) | China | Case-control | Normal colonoscopy | NAA  AA  Ad  CRC  Cn | 36  13  49  31  64 | 58.4  62.4  57.8 | I/ II  III/ IV | 17  13 | qMSP | *SNCA (promoter)* |
| Han, 2019 (26) | South Korea | Case-control | Normal colonoscopy | NAA  0/ AA  Ad  CRC  Cn | 41  6  47  242  245 | __ | I  II  III  IV | 55  70  96  21 | meSDC2 LTE-qMSP | *SDC2 (-)* |
| Liu, 2019 (27) | China | Case-control | Normal colonoscopy | AA  CRC  Cn | 77  80  83 | 61^#^  60^#^  58^#^ | I/ II  III/IV | 43  37 | qMSP | *COL4A1 (promoter/ exon 1)*  *COL4A2* *(promoter/ exon 1)*  *ITGA4 (promoter/ exon 1)*  *TLX2 (promoter/ exon 1)* |

^#^ Median age

* for the combined group including Stage0/AA and CRC

Abbreviations: Ref., reference; No., number; DNAm, DNA methylation; MSP, methylation-specific polymerase chain reaction; qMSP, quantitative methylation-specific polymerase chain reaction; n-MSP, nested methylation-specific polymerase chain reaction; Hi-SA, high-sensitivity assay for bisulfite DNA; LTE, linear target enrichment; MSRH, methylation-specific reverse hybridization; NAA, non-advanced adenoma; AA, advanced adenoma; Ad, adenoma; Cn, Control.

Note- Stages I/II/III/IV as per TNM classification and stages A/B/C/D as per Dukes classification.


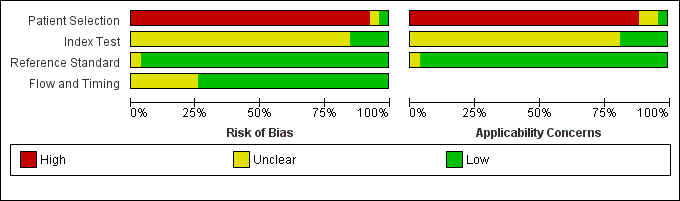


Figure S1. Risk of bias and applicability concerns graph: review authors' judgements about each domain presented as percentages across included studies.


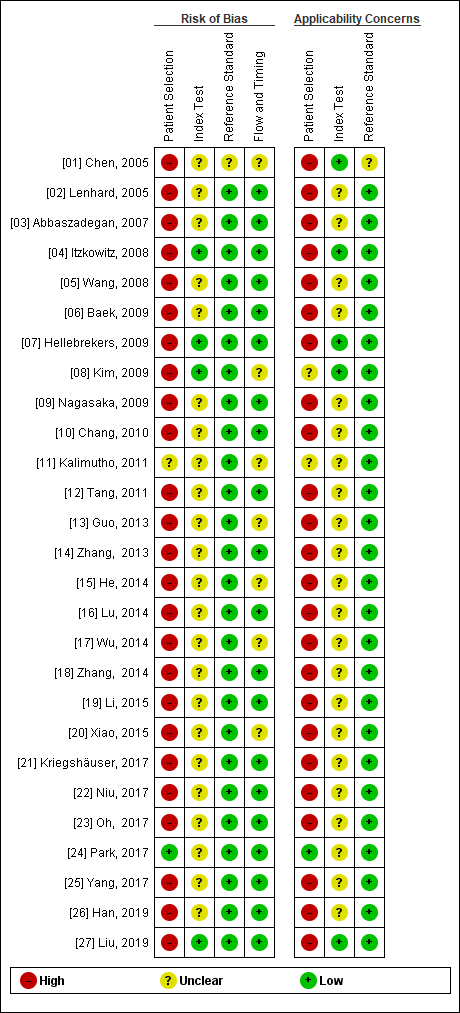


Figure S2. Risk of bias and applicability concerns summary: review authors' judgements about each domain for each included study.

# **References**

1. Chen WD, Han ZJ, Skoletsky J, Olson J, Sah J, Myeroff L, et al. Detection in fecal DNA of colon cancer-specific methylation of the nonexpressed vimentin gene. J Natl Cancer Inst. 2005;97(15):1124-32.

2. Lenhard K, Bommer GT, Asutay S, Schauer R, Brabletz T, Goke B, et al. Analysis of promoter methylation in stool: a novel method for the detection of colorectal cancer. Clin Gastroenterol Hepatol. 2005;3(2):142-9.

3. Abbaszadegan MR, Tavasoli A, Velayati A, Sima HR, Vosooghinia H, Farzadnia M, et al. Stool-based DNA testing, a new noninvasive method for colorectal cancer screening, the first report from Iran. World J Gastroenterol. 2007;13(10):1528-33.

4. Itzkowitz S, Brand R, Jandorf L, Durkee K, Millholland J, Rabeneck L, et al. A Simplified, Noninvasive Stool DNA Test for Colorectal Cancer Detection. American Journal of Gastroenterology. 2008;103(11):2862-70.

5. Wang DR, Tang D. Hypermethylated SFRP2 gene in fecal DNA is a high potential biomarker for colorectal cancer noninvasive screening. World J Gastroenterol. 2008;14(4):524-31.

6. Baek YH, Chang E, Kim YJ, Kim BK, Sohn JH, Park DI. Stool methylation-specific polymerase chain reaction assay for the detection of colorectal neoplasia in Korean patients. Dis Colon Rectum. 2009;52(8):1452-9; discussion 9-63.

7. Hellebrekers DM, Lentjes MH, van den Bosch SM, Melotte V, Wouters KA, Daenen KL, et al. GATA4 and GATA5 are potential tumor suppressors and biomarkers in colorectal cancer. Clin Cancer Res. 2009;15(12):3990-7.

8. Kim MS, Louwagie J, Carvalho B, Terhaar Sive Droste JS, Park HL, Chae YK, et al. Promoter DNA methylation of oncostatin m receptor-beta as a novel diagnostic and therapeutic marker in colon cancer. PLoS One. 2009;4(8):e6555.

9. Nagasaka T, Tanaka N, Cullings HM, Sun DS, Sasamoto H, Uchida T, et al. Analysis of fecal DNA methylation to detect gastrointestinal neoplasia. J Natl Cancer Inst. 2009;101(18):1244-58.

10. Chang E, Park DI, Kim YJ, Kim BK, Park JH, Kim HJ, et al. Detection of colorectal neoplasm using promoter methylation of ITGA4, SFRP2, and p16 in stool samples: a preliminary report in Korean patients. Hepatogastroenterology. 2010;57(101):720-7.

11. Kalimutho M, Di Cecilia S, Del Vecchio Blanco G, Roviello F, Sileri P, Cretella M, et al. Epigenetically silenced miR-34b/c as a novel faecal-based screening marker for colorectal cancer. Br J Cancer. 2011;104(11):1770-8.

12. Tang D, Liu J, Wang DR, Yu HF, Li YK, Zhang JQ. Diagnostic and prognostic value of the methylation status of secreted frizzled-related protein 2 in colorectal cancer. Clin Invest Med. 2011;34(2):E88-95.

13. Guo Q, Song Y, Zhang H, Wu X, Xia P, Dang C. Detection of hypermethylated fibrillin-1 in the stool samples of colorectal cancer patients. Med Oncol. 2013;30(4):695.

14. Zhang H, Song YC, Dang CX. Detection of hypermethylated spastic paraplegia-20 in stool samples of patients with colorectal cancer. Int J Med Sci. 2013;10(3):230-4.

15. He CG, Huang QY, Chen LS, Ling ZA, Wu HG, Deng HQ. p33(ING1b) methylation in fecal DNA as a molecular screening tool for colorectal cancer and precancerous lesions. Oncol Lett. 2014;7(5):1639-44.

16. Lu H, Huang S, Zhang X, Wang D, Zhang X, Yuan X, et al. DNA methylation analysis of SFRP2, GATA4/5, NDRG4 and VIM for the detection of colorectal cancer in fecal DNA. Oncol Lett. 2014;8(4):1751-6.

17. Wu XD, Song YC, Cao PL, Zhang H, Guo Q, Yan R, et al. Detection of miR-34a and miR-34b/c in stool sample as potential screening biomarkers for noninvasive diagnosis of colorectal cancer. Med Oncol. 2014;31(4):894.

18. Zhang H, Zhu YQ, Wu YQ, Zhang P, Qi J. Detection of promoter hypermethylation of Wnt antagonist genes in fecal samples for diagnosis of early colorectal cancer. World J Gastroenterol. 2014;20(20):6329-35.

19. Li WH, Zhang H, Guo Q, Wu XD, Xu ZS, Dang CX, et al. Detection of SNCA and FBN1 methylation in the stool as a biomarker for colorectal cancer. Dis Markers. 2015;2015:657570.

20. Xiao W, Zhao H, Dong W, Li Q, Zhu J, Li G, et al. Quantitative detection of methylated NDRG4 gene as a candidate biomarker for diagnosis of colorectal cancer. Oncology Letters. 2015;9(3):1383-7.

21. Kriegshauser G, Enko D, Zitt M, Oberwalder M, Oberkanins C, Ofner D, et al. Comparison of a prototype reverse hybridization assay and MethyLight for detection of SFRP2 promotor methylation in fecal DNA. Int J Biol Markers. 2017;32(4):e467-e70.

22. Niu F, Wen J, Fu X, Li C, Zhao R, Wu S, et al. Stool DNA Test of Methylated Syndecan-2 for the Early Detection of Colorectal Neoplasia. Cancer Epidemiol Biomarkers Prev. 2017;26(9):1411-9.

23. Oh TJ, Oh HI, Seo YY, Jeong D, Kim C, Kang HW, et al. Feasibility of quantifying SDC2 methylation in stool DNA for early detection of colorectal cancer. Clin Epigenetics. 2017;9:126.

24. Park SK, Baek HL, Yu J, Kim JY, Yang HJ, Jung YS, et al. Is methylation analysis of SFRP2, TFPI2, NDRG4, and BMP3 promoters suitable for colorectal cancer screening in the Korean population? Intest Res. 2017;15(4):495-501.

25. Yang QL, Wang SM, Ma J, Li XW, Liu X, Peng MN, et al. Identification the potential of stool-based SNCA methylation as a candidate biomarker for early colorectal cancer detection. Translational Cancer Research. 2017;6(1):169-+.

26. Han YD, Oh TJ, Chung TH, Jang HW, Kim YN, An S, et al. Early detection of colorectal cancer based on presence of methylated syndecan-2 (SDC2) in stool DNA. Clin Epigenetics. 2019;11(1):51.

27. Liu X, Wen J, Li C, Wang H, Wang J, Zou H. High-Yield Methylation Markers for Stool-Based Detection of Colorectal Cancer. Dig Dis Sci. 2019.
